# Absence of social desirability bias in the evaluation of chronic disease self-management interventions

**DOI:** 10.1186/1477-7525-11-114

**Published:** 2013-07-08

**Authors:** Sandra Nolte, Gerald R Elsworth, Richard H Osborne

**Affiliations:** 1 Medical Clinic for Psychosomatics, Charité - University Medicine Berlin, Charitéplatz 1, 10117 Berlin, Germany; 2 Public Health Innovation, Population Health Strategic Research Centre, Deakin University, School of Health & Social Development, Faculty of Health, 221 Burwood Highway, Burwood VIC 3125, Australia

**Keywords:** Chronic disease, Patient education, Chronic disease self-management, Program evaluation, Structural equation modeling, Social desirability, Statistical bias

## Abstract

**Background:**

Bias due to social desirability has long been of concern to evaluators relying on self-report data. It is conceivable that health program evaluation is particularly susceptible to social desirability bias as individuals may be inclined to present themselves or certain health behaviors in a more positive light and/or appease the course leader. Thus, the influence of social desirability bias on self-report outcomes was explored in the present study.

**Methods:**

Data were collected from 331 participants of group-based chronic disease self-management interventions using the highly robust eight-scale Health Education Impact Questionnaire (heiQ) and the 13-item short form Marlowe-Crowne Social Desirability Scale (MC-C). The majority of self-management courses were run by community-based organizations across Australia between February 2005 and December 2006 where 6 to 12 individuals have the opportunity to develop considerable rapport with course leaders and each other over about six weeks. Pre-test data were collected on the first day of courses, while post-test and social desirability scores were assessed at the end of courses. A model of partial mediation within the framework of structural equation modeling was developed with social desirability as the mediating variable between pre-test and post-test.

**Results:**

The ‘Defensiveness’ factor of the MC-C showed clear association with heiQ pre-test data, a prerequisite for investigating mediation; however, when investigating the eight full pre-test/post-test models ‘Defensiveness’ was only associated with one heiQ scale. This effect was small, explaining 8% of the variance in the model. No other meditational effects through social desirability were observed.

**Conclusions:**

The overall lack of association of social desirability with heiQ outcomes was surprising as it had been expected that it would explain at least some of the variance observed between pre-test and post-test. With the assumption that the MC-C captures the propensity for an individual to provide socially desirable answers, this study concludes that change scores in chronic disease self-management program evaluation are not biased by social desirability.

## Background

There has long been a concern that considerable bias in survey research can stem from respondents providing answers that are partly determined by social influences, in particular social desirability [1]. While the influence of social desirability bias has been found to vary according to the survey method, telephone and personal interviews have been found to be particularly prone to socially desirable responding [1-3]. Hence, social desirability bias may be a major threat to the validity of self-report outcomes data. Although there are several elements to its conceptualization, social desirability bias can generally be described as a response style exhibited by respondents who endorse items that represent traits and/or behaviors that they think stand for a socially acceptable or endorsed position [4]. Further, it can be differentiated between two dimensions: 1) the need for social approval, i.e. creation of a positive impression of oneself to receive approval from others (impression management), and 2) self-deception or defensiveness, i.e. avoidance of disapproval by denying socially undesirable traits and/or behaviors [5-8]. Social desirability has been found to be related to demographic variables; it is more likely to be identified in older women [9,10], women of lower socio-economic status [10,11], and higher age [12,13]. Finally, social desirability has been found to be strongly related to the positive rating of the personal qualities of self, family and friends and not of ‘people in general’, the so-called ‘better than average’ effect [14].

While social desirability bias has been a general concern in evaluations based on self-reports [3], it may play a particularly important role in chronic disease health education interventions, in particular those that are offered to groups of people with chronic conditions who were initially unknown to each other. First, it is likely that individuals would be inclined to present themselves or certain health behaviors in a more positive light. This phenomenon would generally apply to any health-related outcomes assessment. Second, in the specific context of group-based interventions, it is intended that participants and course leaders build strong rapport during the intervention that may last several weeks or months. As a result, at the end of courses, participants may be inclined to provide socially desirable answers to endorse course leaders regardless of whether they truly benefited from the intervention. That is, participants may be aware that they are indirectly evaluating the performance of both the course leader and the organization and therefore provide socially desirable responses to appease leaders rather than showing how they really felt after graduating from the self-management course. Finally, in this setting, participants often fill out questionnaires in the presence of leaders and their peers which again may trigger socially desirable responses as they may feel pressurized to endorse the leaders’ performance. Hence, social desirability bias may have a particular influence on post-test scores and thus apparent change scores.

To measure the influence of potential socially desirable responses, several scales have been developed [5,15-18]. Of these, the Marlowe-Crowne (MC) Social Desirability scale [16] is one of the most widely used indices [19]. It is commonly described as a measure of a person’s need for approval. Although the original authors defined the concept of social desirability in terms of two dimensions, i.e. need for approval and avoidance of disapproval [6,20], they conceptualized the MC scale as a measure of a single dimension [6,21]. However, subsequent studies found little support for this hypothesis, with results ranging from two-factor [5,22] to multi-factor solutions [19,21,23-26]. While such findings cast some doubt on the measurement properties of the MC scale, these studies should be treated with caution. Only two studies applied rigorous psychometric statistical techniques to investigate the properties of the MC scale [19,21]. Moreover, the generalizability of studies is questionable as almost all samples consisted of students [19,24,25,27-29].

The original MC scale consists of 33 items. Therefore, for some respondents it may be a burden to complete, particularly if the scale is among a panel of scales. As a consequence, short forms have been developed, with Reynolds’ (1982) and Strahan and Gerbasi’s (1972) short forms being most frequently applied [19,21]. Commentaries on the usefulness of the short forms vary substantially. While some suggest that all are unsatisfactory [19,24], others show that they are improvements over the original [25,26,28]. However, these studies should also be treated with caution. Apart from one study [19] none applied rigorous statistical methods. Further, factor analyses on the short forms were generally aimed at confirming/rejecting the one-factor hypothesis, whereas none tested the scales for a potential two- or multi-factor solution. Of all short forms, Reynolds’ MC-C [30] has been explored extensively [31] and is one of the most frequently used short forms [32-34]. It has generally been described as a reliable alternative to the full scale [30,31,35] with acceptable internal consistency [24,25,30,31,34].

In summary, social desirability bias has received frequent attention in the literature [20,36]. However, in view of its potential threat to the validity of scores derived from participants of health interventions, it is surprising that this bias has rarely been explored in contexts where social desirability is likely to be an important bias. Only two out of more than 100 controlled trials of chronic disease self-management courses considered social desirability as a potential covariate [37]. The aim of this study was to explore the influence of social desirability bias on change scores derived from data collected from groups of participants taking part in chronic disease self-management courses.

## Methods

### Courses and participants

Data were collected from 331 participants of chronic disease self-management courses implemented mainly by community-based organizations across Australia between February 2005 and December 2006. As shown in Table 1, three quarters of respondents were female (74.2%), mean age 62.2 years (age range 19 to 90 years), and the majority reported to be affected by osteoarthritis (45.5%), depression (29.9%), diabetes (22.1%), and asthma (21.5%). The predominant course type (71.2%) was a generic intervention [38], while the remaining disease-specific interventions were mostly aimed at people with arthritis.

**Table 1:** Demographic characteristics of respondents

	**n = 331**	
	**n**	**%**
Gender		
Female	244	74.2
Male	85	25.8
Age		
Mean (standard deviation)	62.2 (13.2)	
Range	19-90	
Education		
Primary education	31	9.8
Up to year 8	100	31.7
Year 9 to 12	82	26.0
TAFE^1^	59	18.7
University	43	13.7
Employment status		
Full-time	13	4.2
Part-time	21	6.7
Unemployed	28	8.9
Home duties	42	13.4
Retired	204	65.2
Other	5	1.6
Birth place		
Australia	241	73.5
Born elsewhere	87	26.5
Chronic condition (more than one could be selected)		
Asthma	69	21.5
Cancer	17	5.3
Coronary heart disease	42	13.1
Depression	96	29.9
Diabetes	71	22.1
Fibromyalgia	37	11.5
Osteoarthritis	146	45.5
Osteoporosis	47	14.6
Rheumatoid arthritis	51	15.9
Other	140	43.8

^1^*TAFE* Technical and further education.

Participant recruitment was undertaken at a course level where leaders were recruited through established networks and snowball recruitment as previously described [39,40]. Pre-test data were provided at the start of courses (T_1_), while post-test and social desirability data were collected at the end of courses (T_2_), on average six weeks after pre-test. The 13-item short form MC-C was applied [30]. Questions were answered using a ‘true-false’ response scale in the same manner as in the original scale [16]. The Health Education Impact Questionnaire (heiQ), a widely used measure of impacts of self-management interventions, was used to collect patient-reported outcomes data [41,42]. The version of the heiQ that was applied comprised 38 items, each uniquely associated with one of the following eight factors: Positive and active engagement in life, Health directed activities, Skill and technique acquisition, Constructive attitudes and approaches, Self-monitoring and insight, Health service navigation, Social integration and support, and Emotional distress. All items were measured on a 6-point Likert response scale ranging from “strongly disagree” to “strongly agree”.

### Statistical model

As described in the introduction, previous research on the validity of the MC scale lacked both statistical sophistication and samples including people with chronic disease [5,7,19,22,24]. Consequently, it was deemed necessary to determine the psychometric properties of the MC-C before embarking upon the analyses. This was approached in an exploratory way. Data were first analyzed in CEFA [43], a computer program for unrestricted factor analyses [44]. As the MC-C was assumed to measure one underlying construct, i.e. social desirability, multi-factor structures were analyzed with oblique rotation to allow for correlations between factors. For this GEOMIN was used [44,45]. Due to the scaling of the MC-C, the input matrix was based on polychoric correlations and the ordinary least squares method was used for parameter estimation [43]. Once the factor structure was determined, it was again tested in LISREL version 8.72 [46], using Robust Maximum Likelihood (RML), to both confirm the model and estimate model parameters [47].

For evaluation of the model resulting from the confirmatory factor analysis, a combination of fit statistics was chosen for a comprehensive assessment of model fit, i.e. a range of qualitatively different fit statistics was applied [48-50]. First, the χ^2^ statistic [51] was used. It is based on the comparison of the model covariance matrix with the sample covariance matrix. If a non-significant χ^2^ is obtained, this indicates that the two matrices do not differ significantly, i.e. it indicates that the model fits well [52]. Second, the root mean square error of approximation (RMSEA) was chosen, with values of < 0.05 indicating close fit and those of < 0.08 indicating acceptable fit [53]. Third, for the standardized root mean square residuals (SRMR) a value of up to 0.08 was considered acceptable. Finally, the comparative fit index (CFI) was selected, with a cut-off value of 0.95 or above [54,55].

In a second step, a model of partial mediation was developed in the framework of structural equation modeling (SEM) again using LISREL [46]. Social desirability was included as a mediating variable between predictor (pre-test) and outcome (post-test) following Kenny and colleagues [56-58]. To establish whether social desirability was a mediator between heiQ pre-test and post-test data, the following conditions had to be established [56,57]:

1) Mediator and predictor must correlate, i.e. the predictor must affect the mediating variable for the latter to be a mediator between predictor and outcome. This was tested by regressing mediator (MC-C) on predictor (heiQ pre-test).

2) The predictor must affect the outcome. This was tested by regressing outcome (heiQ post-test) on predictor (heiQ pre-test).

3) The mediator must affect the outcome, i.e. it had to be established that the regression of outcome on mediator was significant. In this model, MC-C was included as a second endogenous variable, i.e. both heiQ post-test and MC-C were regressed on heiQ pre-test.

4) Once conditions (1) to (3) were met, the statistical significance of the mediational effect was tested, i.e. the statistical significance of the product of the paths from a) predictor to mediator, and b) mediator to outcome [59-61].

5) Finally, while steps (1) to (4) are both necessary and sufficient conditions to establish mediation, the mediational effect must be interpreted in the overall context of the model [61]. Thus, it was assessed what proportion of the total effect was being mediated.

**Figure 1:**
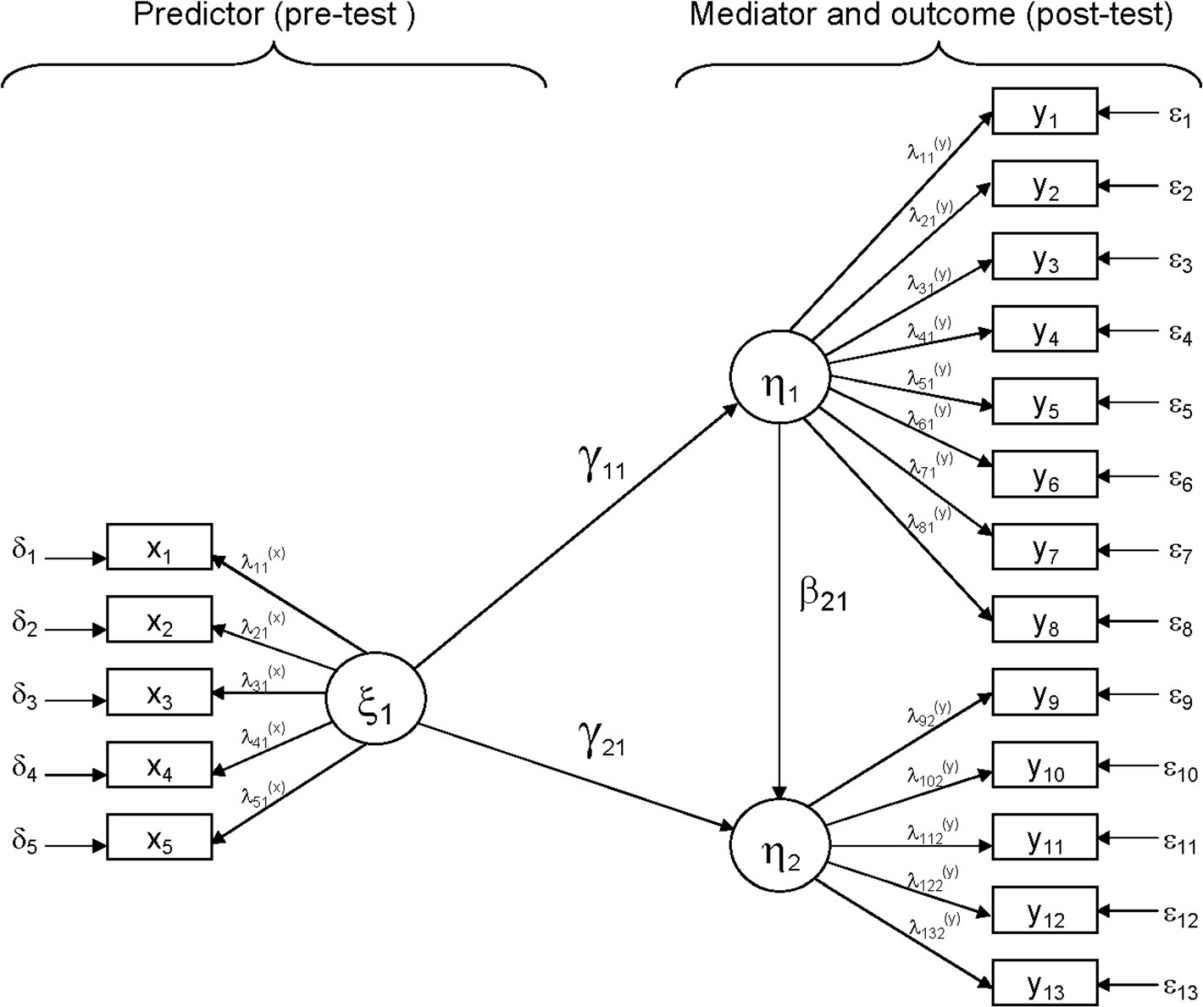
Structural equation model, following LISREL notation, with the short form Marlowe-Crowne social desirability scale MC-C as a partial mediating variable.

An example of the model using one hypothetical heiQ scale is visualized in Figure 1 where both MC-C and heiQ post-test are regressed on heiQ pre-test, and heiQ post-test is regressed on the MC-C.

Before analyzing heiQ and MC-C data, some preparatory steps were undertaken. First, each case with more than 50% missing items was deleted. Second, due to the alternate keying of the MC-C items, it could easily be detected if participants exhibited an acquiescent response style [62]. Consequently, respondents who had provided either only ‘true’ or only ‘false’ answers were discarded. It was assumed that they had filled out the MC-C regardless of item content. Once this preparation was finalized, all remaining missing values were replaced using the EM Algorithm [63], leading to a final sample size of n = 318.

## Results

Exploratory factor analyses of the MC-C using CEFA suggested that a one-factor solution did not fit the data well. With two eigenvalues clearly above one (3.4 and 1.9, respectively) and two further eigenvalues at 1.1, factor solutions ranging between two factors and four factors were explored. While fit statistics improved in all multi-factor solutions, models beyond two factors were not superior to the two-factor solution. Therefore, a two-factor solution – labeled SD1 ‘defensiveness’ and SD2 ‘self-presentation’ – appeared most suitable for the MC-C with a moderate correlation of the two factors (0.48). As shown in Table 2, this solution was confirmed in LISREL. While fit statistics were excellent (non-significant Satorra-Bentler chi-square [64,65], RMSEA = 0.023 [90% CI, 0.0;0.043], CFI = 0.99, and SRMR = 0.079), some small factor loadings were obtained ranging from 0.33 to 0.76. Reliability was also relatively low, with Coefficient alpha at 0.59 for SD1 and 0.56 for SD2. As the validation of the MC-C was of exploratory nature [54], these values were deemed acceptable for the present study.

**Table 2:** Confirmatory factor analysis of the short form Marlowe-Crowne social desirability scale MC-C (n = 318)

		**Factor loading**	**Error variance**
Self-presentation			
1	It is sometimes hard for me to go on with my work if I am not encouraged.	0.44	0.81
2	I sometimes feel resentful when I don’t get my way.	0.76	0.43
3	On a few occasions, I have given up doing something because I thought too little of my ability.	0.51	0.74
4	There have been times when I felt like rebelling against people in authority even though I knew they were right.	0.54	0.71
6	There have been occasions when I took advantage of someone.	0.33	0.89
8	I sometimes try to get even rather than forgive and forget.	0.48	0.77
11	There have been times when I was quite jealous of the good fortune of others.	0.51	0.74
12	I am sometimes irritated by people who ask favours of me.	0.37	0.87
Defensiveness			
5	No matter who I’m talking to, I’m always a good listener.	0.54	0.71
7	I’m always willing to admit it when I make a mistake.	0.64	0.59
9	I am always courteous, even to people who are disagreeable.	0.70	0.51
10	I have never been irked when people expressed ideas very different from my own.	0.48	0.77
13	I have never deliberately said something that hurt someone’s feelings.	0.42	0.83

Fit statistics: χ^2^_SB_(63) = 73.7, *p* = NS; RMSEA = 0.023 (90% CI, 0.0;0.043); CFI = 0.99; SRMR = 0.079. Coefficient alpha: factor 1 = 0.59; factor 2 = 0.56; Phi = 0.48.

### Social desirability in heiQ data

The first step of the 5-step procedure suggested that ‘defensiveness’ correlated significantly with pre-test data across all heiQ scales. Correlations ranged from 0.24 to 0.39, equivalent to a small to medium effect [59,66]. In contrast, none of the heiQ scales indicated an association between ‘self-presentation’ and pre-test data (Table 3). Thus, only ‘defensiveness’ was explored as a potential partial mediating variable in heiQ data, while ‘self-presentation’ could be ruled out as a mediator.

**Table 3:** Covariance between ‘defensiveness’, ‘self-presentation’, and heiQ pre-test data

		**HDA**	**PAE**	**ED**	**SMI**	**CAA**	**STA**	**SIS**	**HSN**
SD1-SD2	Cov	0.254*	0.247*	0.251*	0.249*	0.248*	0.246*	0.250*	0.240*
	(SE)	(0.060)	(0.058)	(0.059)	(0.058)	(0.058)	(0.058)	(0.059)	(0.058)
	Corr	0.482	0.480	0.473	0.478	0.475	0.477	0.480	0.477
SD1-pre	Cov	0.208*	0.192*	0.233*	0.312*	0.360*	0.255*	0.264*	0.199*
	(SE)	(0.071)	(0.071)	(0.069)	(0.092)	(0.084)	(0.057)	(0.079)	(0.057)
	Corr	0.255	0.242	0.280	0.310	0.388	0.375	0.280	0.271
SD2-pre	Cov	0.089	0.015	−0.032	0.155	0.032	0.098	0.009	0.019
	(SE)	(0.068)	(0.068)	(0.065)	(0.098)	(0.076)	(0.061)	(0.070)	(0.066)
	Corr	0.116	0.020	−0.041	0.156	0.037	0.153	0.010	0.026

* Significant, i.e. the effect is more than twice its standard error (Bollen, 1989).

Legend

*HDA* Health-directed activity.

*PAE* Positive and active engagement in life.

*ED* Emotional distress.

*SMI* Self-monitoring and insight.

*CAA* Constructive attitudes and approaches.

*STA* Skill and technique acquisition.

*SIS* Social integration and support.

*HSN* Health service navigation.

*SD1* Factor 1 ‘defensiveness’.

*SD2* Factor 2 ‘self-presentation’.

*Cov* Covariance.

*SE* Standard error.

*Corr* Correlation.

In Step 2 it was found that all direct paths from pre-test to post-test were significant. While subscale Social integration and support showed the strongest association between the two scores, all heiQ subscales showed substantial paths from predictor to outcome (Table 4).

**Table 4:** Regression of heiQ post-test on heiQ pre-test datav

		**HDA**	**PAE**	**ED**	**SMI**	**CAA**	**STA**	**SIS**	**HSN**
pre-post	Path	0.648*	0.829*	0.758*	0.593*	0.672*	0.610*	0.774*	0.743*
	(SE)	(0.044)	(0.069)	(0.049)	(0.071)	(0.066)	(0.065)	(0.044)	(0.053)
	stand.	0.744	0.776	0.761	0.665	0.718	0.626	0.808	0.777

* Significant, i.e. the effect is more than twice its standard error (Bollen, 1989).

Legend

For an extensive legend refer to Table 3.

Finally, Table 5 presents the associations between pre-test and post-test once SD1 was included in the models. Again, paths between pre-test and ‘defensiveness’ were significant. Once pre-test data were controlled for ‘defensiveness’, Emotional distress was the only subscale that showed a significant association between ‘defensiveness’ and heiQ post-test data.

**Table 5:** Regression of ‘defensiveness’ and heiQ post-test data on heiQ pre-test data, and regression of heiQ post-test data on ‘defensiveness’

		**HDA**	**PAE**	**ED**	**SMI**	**CAA**	**STA**	**SIS**	**HSN**
pre-post	Path	0.654*	0.826*	0.696*	0.575*	0.698*	0.606*	0.765*	0.757*
	(SE)	(0.049)	(0.072)	(0.052)	(0.077)	(0.076)	(0.075)	(0.046)	(0.058)
	stand.	0.753	0.772	0.700	0.642	0.752	0.618	0.796	0.788
pre-SD1	Path	0.172*	0.169*	0.191*	0.166*	0.229*	0.301*	0.160*	0.192*
	(SE)	(0.057)	(0.059)	(0.056)	(0.049)	(0.052)	(0.067)	(0.047)	(0.053)
	stand.	0.259	0.252	0.290	0.326	0.399	0.388	0.284	0.283
SD1-post	Path	−0.038	0.026	0.322*	0.128	−0.141	0.024	0.070	−0.060
	(SE)	(0.088)	(0.107)	(0.099)	(0.132)	(0.123)	(0.101)	(0.092)	(0.102)
	stand.	−0.029	0.016	0.213	0.073	−0.087	0.019	0.041	−0.043

* Significant, i.e. the effect is more than twice its standard error (Bollen, 1989).

Legend

For an extensive legend refer to Table 3.

As ‘defensiveness’ was found to be associated with Emotional distress, steps 4 and 5 were performed on this heiQ scale only. Once ‘defensiveness’ was included in the model, the path between pre-tests and post-tests decreased by 0.062, a significant effect, as it was more than twice its standard error [52], i.e. SE_M_ = √ (0.191^2^ * 0.099^2^ + 0.322^2^ * 0.056^2^) = 0.026. The magnitude of the effect, however, was small as it contributed only 8.2% of the total variation in change scores.

## Discussion

This study explored the potential mediating effect of social desirability in the measurement of outcomes of chronic disease self-management courses. For this, we used rigorous statistical techniques – including exploratory and confirmatory factor analysis of the MC-C as well as applying a comprehensive 5-step model – to explore both direct and mediating effects on key outcomes. Surprisingly, while we had expected clear evidence of bias in estimates of change through socially desirable responding, virtually no social desirability bias was found. When analyzing social desirability bias as a potential mediating variable between heiQ pre-test and post-test data, only the ‘defensiveness’ factor but not the ‘self-presentation’ factor of the MC-C showed an association with pre-test data, a prerequisite for investigating mediation. The notion of ‘defense’ and ‘self-protection’ was introduced as one critical aspect of the approval motive [6]. Subsequent research, however, suggested that subjects’ motivations to present themselves in a socially desirable way was linked more strongly to ‘defensiveness’ rather than ‘self-presentation’ [7,67] which may explain our findings, i.e. the lack of association of pre-test data with ‘self-presentation’.

Despite the significant association of ‘defensiveness’ with all pre-tests, it exerted only little influence on heiQ post-test data once pre-test data were controlled for. Only one heiQ scale (Emotional distress) showed that ‘defensiveness’ operated as a true, albeit minor, mediator. Therefore, the influence of social desirability bias in heiQ data can largely be ruled out. This finding is contrary to our expectations. First, the specific context of group-based chronic disease self-management interventions, potential rapport among participants and between participants and course leader(s), and provision of data in the presence of course leaders are factors that may be conducive to exhibiting a socially desirable response style. Second, social desirability has been found to be related to a range of demographic variables. Among others, older women [9,10], women of lower socio-economic status [10,11], and older respondents [12,13] have been found to be most prone to socially desirable responding. While we did not have socio-economic data, the remaining characteristics largely fit our sample, i.e. an additional argument for the presence of social desirability in our study.

There are several possible explanations for our findings, i.e. lack of social desirability bias in heiQ data. First, all heiQ items have been written in a way that discourage response styles [41]. That is, even people who are usually prone to socially desirable responding may have been discouraged to do so through the content and structure of heiQ items. The heiQ was developed using grounded approaches including the use of concepts and wording that were directly derived from patients. Second, the short form MC-C scale was used to explore a potential effect of social desirability bias. Although there is sufficient support in the literature that the MC-C is a valid alternative to the full MC scale, and our re-validation supported a two-factor solution with excellent fit statistics, it is possible that the analyses were hampered by a suboptimal performance of this shortened measure. Despite excellent fit indices in LISREL, low reliability and some small factor loadings may have limited the power of the analyses to detect mediational effects of social desirability.

In this study we applied a novel approach to testing the influence of social desirability bias in the context of chronic disease self-management programs. Apart from providing a detailed re-validation of the MC-C [30], with both exploratory and confirmatory analyses, a sophisticated model of partial mediation was developed that should have detected an association of social desirability if there had been any. However, it cannot be ruled out that the MC-C scale did not perform sufficiently well, while a potential co-existence of equivalent models also needs to be acknowledged [68,69]. For example, it would have been plausible to define ‘defensiveness’ as a predictor of both pre-test and post-test or define a model of moderated mediation [57,59], with variables such as age, gender, or education operating as moderating variables. It is possible that there was a mediating effect of socially desirable responding in older participants but not in their younger counterparts. The sample size of the dataset, however, did not allow for such modeling. Further, it is possible that social desirability moderated – rather than mediated – the effect between pre-test and post-test. However, as ‘social desirability’ was defined as a response style that was hypothesized to improve the prediction of post-test levels – i.e. the variable ‘social desirability’ was defined as part of the causal chain [56,57] – current model definition was assumed to be most appropriate to test for socially desirable responding. In view of our specific research questions, the present model is a logical and theoretically sound approach [61]. That is, the path between pre-test and post-test was understood as the primary path in the model, and social desirability was defined as a response style that potentially partially mediated the relationship between heiQ pre-test and post-test data.

## Conclusions

The analyses of this study also provided support for the measurement qualities of the heiQ. That is, data derived from this questionnaire appear robust against bias through socially desirable responding. Based on the present research, the use of the heiQ within the traditional method of assessing change (post-test minus pre-test) appears immune to potential confounding effects through social desirability. However, further research is necessary to ascertain whether this bias is present at the subject-level. To advance the field, a combination of qualitative and quantitative approaches at group-level and individual-level is needed and questionnaires other than Reynold’s short-form should be used to further explore whether social desirability bias exists in the evaluation of chronic disease self-management programs. With the assumption that Reynold’s short-form of the Marlowe-Crowne Social Desirability scale captures the propensity for individuals to provide socially desirable answers, change scores in patient education program evaluation are not biased by social desirability.

## Ethical adherence

The study was approved by the Human Research Ethics Committee of the University of Melbourne.

## Abbreviations

heiQ: Health education impact questionnaire; MC: Marlowe-Crowne Social Desirability scale; MC-C: 13-item short form Marlowe-Crowne Social Desirability scale; RML: Robust maximum likelihood; SEM: Structural equation modeling.

## Competing interests

The authors state that there are no conflicts of interests.
